# A multi-analyte serum test for the detection of non-small cell lung cancer

**DOI:** 10.1038/sj.bjc.6605865

**Published:** 2010-09-21

**Authors:** E C Farlow, M S Vercillo, J S Coon, S Basu, A W Kim, L P Faber, W H Warren, P Bonomi, M J Liptay, J A Borgia

**Affiliations:** 1 Department of General Surgery, Rush University Medical Center, 785 Jelke-Southcenter, 1750 W. Harrison Street, Chicago, IL 60612, USA; 2 Department of Pathology, Rush University Medical Center, 532 Jelke-Southcenter, 1750 W. Harrison Street, Chicago, IL 60612, USA; 3 Department of Preventative Medicine, Rush University Medical Center, 470 Triangle Office Building, 1700 W. Van Buren Street, Chicago, IL 60612, USA; 4 Department of Thoracic Surgery, Rush University Medical Center, 774 Profession Office Building, 1725 W. Harrison Street, Chicago, IL 60612, USA; 5 Department of Medical Oncology, Rush University Medical Center, 821 Professional Office Building, 1725 W. Harrison Street, Chicago, IL 60612, USA; 6 Department of Biochemistry, Rush University Medical Center, 558 Cohn Research Building, 1735 W. Harrison Street, Chicago, IL 60612, USA

**Keywords:** Luminex, serum, non-small cell lung cancer, diagnosis

## Abstract

**Background:**

In this study, we appraised a wide assortment of biomarkers previously shown to have diagnostic or prognostic value for non-small cell lung cancer (NSCLC) with the intent of establishing a multi-analyte serum test capable of identifying patients with lung cancer.

**Methods:**

Circulating levels of 47 biomarkers were evaluated against patient cohorts consisting of 90 NSCLC and 43 non-cancer controls using commercial immunoassays. Multivariate statistical methods were used on all biomarkers achieving statistical relevance to define an optimised panel of diagnostic biomarkers for NSCLC. The resulting biomarkers were fashioned into a classification algorithm and validated against serum from a second patient cohort.

**Results:**

A total of 14 analytes achieved statistical relevance upon evaluation. Multivariate statistical methods then identified a panel of six biomarkers (tumour necrosis factor-*α*, CYFRA 21-1, interleukin-1ra, matrix metalloproteinase-2, monocyte chemotactic protein-1 and sE-selectin) as being the most efficacious for diagnosing early stage NSCLC. When tested against a second patient cohort, the panel successfully classified 75 of 88 patients.

**Conclusions:**

Here, we report the development of a serum algorithm with high specificity for classifying patients with NSCLC against cohorts of various ‘high-risk’ individuals. A high rate of false positives was observed within the cohort in which patients had non-neoplastic lung nodules, possibly as a consequence of the inflammatory nature of these conditions.

Lung cancer remains the second most diagnosed cancer in the United States and the most common cause of cancer mortality, with an estimated 161 000 deaths in 2008, with 80% being non-small cell lung cancer (NSCLC) (Mulshine and Sullivan, 2005). Although the overall prognosis for patients with lung cancer is poor with a 5-year survival of <15%, patients diagnosed with early stage disease have a much more favourable prognosis. Patients with pathological Stages I and II disease have 5-year survivals of 57–67% and 38–55%, respectively (Lu *et al*, 2004; Singhal *et al*, 2005). Unfortunately, over half of patients with NSCLC present only after metastasis to lymph nodes or distant sites because of its asymptomatic nature at early stages (Lu *et al*, 2004; Singhal *et al*, 2005; Wardwell and Massion, 2005). Therefore, the best prospect for reducing lung cancer mortality remains earlier detection, when surgery may be curative (Lu *et al*, 2004; Singhal *et al*, 2005). A screening tool capable of early stage detection may allow for decreased lung cancer mortality.

Although accepted screening programmes for breast, colon, prostate and cervical cancer have been developed with subsequent decreases in overall disease mortality, lung cancer-screening programmes remain in the research realm (McWilliams and Lam, 2005). There are currently no established methods for screening individuals at high risk for lung cancer that have been proven to reduce mortality (MacMahon *et al*, 2005; Wardwell and Massion, 2005; Gleeson, 2006). Therefore, screening for NSCLC is not currently recommended by any major medical association. Without a nationally defined screening protocol, there is wide variability in the detection and the initiation of treatment for lung cancer (Yorio *et al*, 2009). Since the 1950s, numerous screening methods have been evaluated for this purpose, including chest X-ray, sputum cytology, bronchoscopic procedures, low-dose spiral computed tomography and molecular diagnosis through nucleic acid or protein biomarkers. These modalities have been evaluated both alone and in several combinations. Even though no screening study for lung cancer has proven efficacy in reducing mortality, several of these strategies have improved our understanding of lung cancer progression and allowed for the development of potential future screening and treatment modalities. One of the most promising combinations of these methodologies consists of low-dose spiral computer tomography (CT) with a companion serum test (Ashton and Jett, 2005; Wardwell and Massion, 2005). A spiral CT differs from conventional CT in that it involves continuous motion of the patient through the machine, which results in a quicker examination and better visualisation of internal structures, such as blood vessels and tissues.

Recent advancements in low-dose spiral CT technology have made improvements towards the detection of NSCLC, but its ability to reduce mortality from NSCLC has yet to be established (Ashton and Jett, 2005; Wardwell and Massion, 2005; Gleeson, 2006). With the relatively high cost of spiral CT, the high rate of false positives leading to unnecessary biopsy or surgery, and the need for serial measurements to confirm non-neoplastic disease, addition of an economical serum test to the CT-screening protocol could improve specificity and cost effectiveness. A serum test could be used as an initial screen to assess NSCLC risk, and select for a smaller population that requires further screening with spiral CT. Alternately, a serum test might also be useful in discriminating between non-neoplastic disease and malignancy for a questionable nodule found by CT, thereby eliminating the need for serial CTs or invasive biopsy. We have already successfully validated a biomarker panel with significant sensitivity and specificity for more accurately defining pre-operative nodal status in NSCLC, and we hope to bring a similar diagnostic tool into the screening realm (Borgia *et al*, 2009).

In this study, we have selected an array of 47 candidate biomarkers implicated in NSCLC and screened a total of 135 patients (*n*=90 NSCLC; *n*=43 controls) to evaluate whether we can identify a panel of biomarkers with significant test performance characteristics for differentiating between patients with early stage NSCLC and our control population. We selected candidate biomarkers based on reports in the literature for having value in discriminating NSCLC from control populations (Hatzakis *et al*, 2002; Neuner *et al*, 2002; Molina *et al*, 2003; Kaya *et al*, 2004; Onn *et al*, 2004; Pujol *et al*, 2004; Boldrini *et al*, 2005; Huang *et al*, 2005; Tarro *et al*, 2005; Vielh *et al*, 2005; D’Amico *et al*, 2006; Kaminska *et al*, 2006; Xi *et al*, 2006). Our principal objective was to develop a multi-analyte blood test capable of screening for NSCLC either as a stand-alone diagnostic measure or as a companion test for current CT-based-screening protocols.

## Materials and methods

### Patient populations

Serum specimens were obtained from 90 NSCLC patients as well as two different groups of controls (*n*=43) to approach the complexity that ‘high-risk’ populations pose to a diagnostic measure of this type. All NSCLC patients and controls were obtained in full compliance with the Institutional Revue Board at Rush University Medical Center (RUMC), including formal written consent. Diagnosis confirmation for the NSCLC cohort was obtained from surgical pathology reports on tissue gathered from tumour resection with lymph node dissections. Criteria for study inclusion in the NSCLC cohort were broad (consisted of having a surgical resection with pathological evaluation) and were not limited to any demographic or clinical factor. Control specimens (*n*=31) were obtained from the Department of Rheumatology RUMC and were all involved in a study of osteoarthritis progression. This cohort was selected on the basis of similar demographic characteristics (with respects to age and sex) and had a diagnosed condition with an inflammatory component. A total of 7 out of the 31 patients had a significant smoking history. At the time of specimen accrual, and in clinical follow-up data, these patients had no evidence of any pulmonary disorders or carcinomas of any type. The ‘non-neoplastic disease after surgery’ group consisted of 12 patients with granulomas, pneumonitis or pneumonia. These patients underwent resection secondary to concern for cancer or persistent symptoms after conservative management.

The specimens used for panel validation consisted of the following cohorts: an NSCLC cohort (*n*=33 total) consisting of 25 Stage I, 7 Stage II and 1 Stage III NSCLC patients, all collected at RUMC. A second control cohort of 15 non-neoplastic lung disease patients with surgically resected ‘questionable’ lesions (all from RUMC), and a ‘non-neoplastic disease without surgery’ group consisting of 40 patients with chronic obstructive pulmonary disease (COPD) or asthma were also used in the validation studies. Patients from this COPD/asthma group were seen clinically based on complaints of cough development or change in respiratory symptoms; serum was collected immediately preceding bronchoscopy and CT imaging was then used to evaluate for the presence of pulmonary nodules. The specimens were generously provided by Abbott Laboratories (Abbott Park, IL, USA) without any patient identifiers or clinical follow-up data beyond that associated with serum acquisition. The overall COPD/asthma cohort from which these cases were selected possessed a smoking history similar to the NSCLC cohort (median value of 40 pack years). Phlebotomy protocols and methods for serum preparation for both of these groups were consistent with those we previously described (Borgia *et al*, 2009).

### Collection and storage of serum specimens

Peripheral blood collected at RUMC was obtained from each patient immediately before treatment initiation using standard phlebotomy techniques, with all samples handled and processed in an identical manner, as previously described (Borgia *et al*, 2009). No specimens were subjected to more than two thaw cycles for this study. Control sera were collected in an identical manner and processed as described above.

### Measurement of serum biomarker concentrations

Whenever possible, the Luminex xMAP immunoassay platform was used to measure the circulating levels of biomarkers reported in this report, with ELISA-based immunoassays encompassing only 2 out of the 47 biomarkers tested. These were all performed according to the manufacturer's suggested protocols and were conducted in the following groupings at the Rush Biomarkers and Proteomics Core Facility: C-reactive protein (CRP) and serum amyloid A (Millipore, Billerica, MA, USA); interleukin-1*β* (IL-1*β*), IL-1ra, IL-6, IL-8, IL-10, tumour necrosis factor-*α* (TNF-*α*) and transforming growth factor-*α* (TGF-*α*) (Millipore); IL-2, IL-13, interferon-*γ* (IFN-*γ*), IFN-inducible protein 10 and granulocyte monocyte colony-stimulating factor (GM-CSF) (Bio-Rad Laboratories, Hercules, CA, USA); IL-1*α*, IL-2R*α*, M-CSF, stem cell-derived factor 1*α* (SDF-1*α*) and stem cell factor (Bio-Rad Laboratories); sE-selectin, sP-selectin and soluble intracellular adhesion molecule 1 (R & D systems, Minneapolis, MN, USA); matrix metalloproteinase-2 (MMP-2), MMP-3, MMP-9 and MMP-13 (R & D Systems); death receptor 5 (DR5), tissue necrosis factor – receptor I (TNF-RI) and TNF-RII (Invitrogen, Carlsbad, CA, USA); RANTES, macrophage inflammatory protein-1*α* (MIP-1*α*), MIP-1*β*, monocyte chemotactic protein-1 (MCP-1) and eotaxin (Invitrogen); granulocyte colony-stimulating factor, epidermal growth factor, vascular endothelial growth factor and basic fibroblast growth factor (Invitrogen). In addition, sEGFR (erb-b1), Her-2 (erb-b2), CA125, CA15-3, CA19-9, CEA and CYFRA 21.1 were measured at the University of Pittsburgh Cancer Institute's Luminex Core Facility (Dr Anna E Lokshin, Director) on a fee-for-service basis. All biomarker concentrations were calculated through a five-parametric curve fit as part of the BioPlex Suspension Array System Software v4.0 (Bio-Rad Laboratories). Measurements of TIMP-1 and osteopontin concentrations were conducted using commercially available ELISA assays and in accordance to the kit directions (R & D Systems). Data were collected on a BioTek PowerWave XS plate reader using KC Junior (v1.40.3) software package. A four-parametric curve fit was used to calculate the concentrations from the raw absorbance readings. All assays performed for this study were conducted in a blinded manner and were statistically processed by different personnel to minimise operator bias.

Validation studies used the identical commercially available kits for 14 of the analytes evaluated, following manufacturer's instructions in the following groupings: CRP (Millipore); IL-1ra, IL-6, IL-10, and TNF-*α* (Millipore); IFN-*γ* (Bio-Rad Laboratories); IL-2R*α* (Bio-Rad Laboratories); sE-selectin and sP-selectin (R & D systems); MMP-2 (R & D Systems); MIP-1*α*, MCP-1, and eotaxin (Invitrogen); CA125 and CYFRA 21.1 was again were measured at the University of Pittsburgh Cancer Institute's Luminex Core Facility. The data was collected in the same manner and a five-parametric curve fit was use to calculate the concentrations from the raw absorbance readings.

### Statistical methods

#### Individual biomarker evaluation

Using SPSS 15.0 for Windows (SPSS Inc., Chicago, IL, USA), descriptive statistics (median, range) and graphical displays (histogram, box plot, normal probability plot) for concentrations of each biomarker were obtained. When using manufacturer's suggested dilution factors, data sets with <66% of the total values within the range of the assay were discarded (arbitrary threshold), whereas data sets with only a small portion of the data missing (because of values being reproducibly immediately below the assay range) had missing values either extrapolated or the lowest measured value used in its place. Overall, the data exhibited a departure from normal statistical distributions and, therefore, the Mann–Whitney rank sum test was used to assess the differences in biomarker concentrations between any of the groups. A threshold for significance was set to *P⩽*0.05. Receiver operating characteristic (ROC) curves for predicting patient's lung cancer was also calculated for each individual biomarker, with the criteria for relevance set to an area under the curve (AUC) value of ⩾0.65.

#### Multivariate analysis

The multivariate analysis was performed on an initial panel of biomarkers selected based on univariate analysis. The inclusion criteria for the individual biomarkers in the initial panel was a Mann–Whitney rank sum (two-sided test) *P*-value <0.05 or an area under the ROC curve (AUC) >0.65; the goal was to include all candidate markers of potential value. The multivariate analysis resulted in a final multivariate panel of biomarkers selected from the initial candidate panel based on statistical variable selection performed within the Random Forests package in R (Breiman *et al*, 1984; Breiman, 2001). This use of Random Forests has been previously described in detail by our group (Borgia *et al*, 2009).

The final multivariate panel of biomarkers resulting from the Random Forest variable selection process was then used by a Classification and Regression Tree (CART) algorithm to model a classification tree that predicts NSCLC diagnosis (yes/no) of each patient based on his/her biomarker panel. This analysis was performed using the RPART package of the R statistical software suite (Team, 2007). The predicted probability of a patient having NSCLC from the classification tree was then compared with the pathology-based NSCLC diagnosis to obtain sensitivity and specificity across a range of cut points for decision rules and the resulting ROCs curve.

## Results

### Analysis of individual serum biomarkers according to diagnostic value

Our initial selection consisted of an array of 47 biomarkers; they were selected based either on published reports for each biomarker showing value for at least one of the following functions: NSCLC diagnosis, staging or prognosis (Hatzakis *et al*, 2002; Neuner *et al*, 2002; Molina *et al*, 2003; Kaya *et al*, 2004; Onn *et al*, 2004; Pujol *et al*, 2004; Boldrini *et al*, 2005; Huang *et al*, 2005; Tarro *et al*, 2005; Vielh *et al*, 2005; D’Amico *et al*, 2006; Kaminska *et al*, 2006; Xi *et al*, 2006) or involvement in biological processes implicated in disease progression. The levels of these markers were evaluated in sera from 90 NSCLC patients treated at RUMC and 43 non-cancer controls. Table 1 shows the clinical and pathological characteristics of patients. Several biomarkers, including IL-1*α*, IL-1*β*, IL-2, IL-15, GM-CSF, TGF-*α*, DR5, MMP-13, had a significant portion of their measurements fall below the threshold of assay range (using the manufacturer's suggested dilution factor) and were disqualified from further analysis. These biomarkers exhibited no apparent trends in the raw data warranting reanalysis.

Serum concentrations of TNF-*α*, CYFRA 21.1, IL-1ra, IL-6, IFN-*γ*, IL-2R*α* and CA125 were found to be significantly higher in the NSCLC group (Mann–Whitney rank sum (two-sided) test *P*-values ⩽0.001), whereas the concentration of MCP-1, CRP, MMP-2 and sE-selectin were found to be significantly higher in the control group (*P*-values ⩽0.001). Using a significance threshold of a Mann–Whitney rank sum (two-sided) test *P-*value <0.05 or analysis of the ROC curve ‘AUC’ >0.65, a total of 14 biomarkers were found to be suitable to undergo multivariate analysis. A list of these biomarkers along with the statistical parameters for each is included in Table 2. No significant differences were observed upon examination of biomarker levels associations with age, smoking history and fasting status (all *P*-values were >0.1).

### Classifications based on a multi-analyte panel for identifying early detection of NSCLC

**Figure 1:**
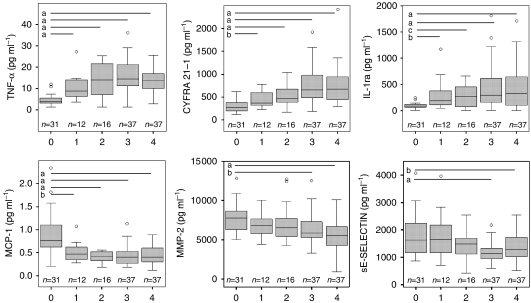
Box plots for the 6 biomarkers identified by the Random Forest algorithm. Box plots for the six selected biomarkers selected by the Random Forest analysis on the discovery cohort. Abscissa labels: 0=surgically resected, non-neoplastic nodules, 1=‘normal’ controls, 2=Stage IA NSCLC, 3=Stage IB NSCLC and 4=Stages II and III (node positive) NSCLC. Notes: disease staging is based on pathologic stage; extreme values are not shown in the plots. Significance (Mann–Whitney Rank sum test) is shown with bars above boxes with a=*P*< 0.001; b=*P*<0.01 and c=*P*<0.05.

**Figure 2:**
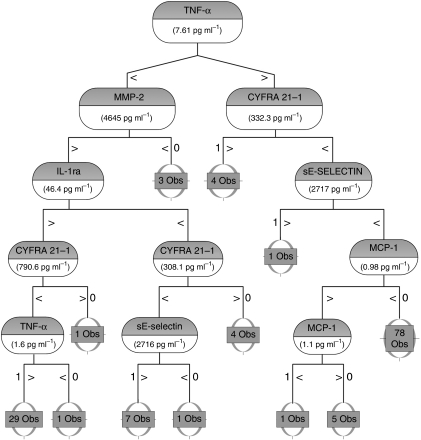
Classification and Regression Tree for Final Panel. Classification and Regression Tree for predicting whether a patient is positive for NSCLC. Briefly, the algorithm represents a series of binary ‘*if-then*’ decision rules that are used to split the data into separate branches of the tree. Each node of the tree displays the analyte being considered and the threshold concentrations used to partition the patient groups. Additional classifications continue along each arm of the split in which it is indicated whether the measured value is either less than or equal to or exceeding the indicated threshold cutoff value. The number of classifications (observations) are listed at each terminal node, with each final arm labelled (0=NSCLC negative; 1=NSCLC positive). Abbreviations: obs.=observations; TNF-*α*=tumour necrosing factor-*α*; MCP-1=monocyte chemotactic protein-1; MMP-2=matrix metalloproteinase-2 and IL-1ra=interleukin-1 receptor antagonist.

**Figure 3:**
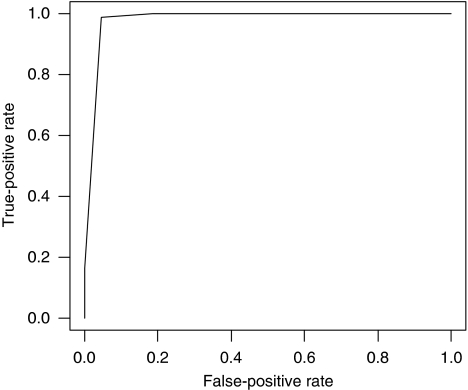
The ROC curve for the six-analyte serum test. ROC curve for the optimised six-analyte CART algorithm using the original training cohort of patients. Area under the curve=0.979; sensitivity=99% specificity=95%.

A panel of 6 biomarkers was selected from the 14 biomarkers meeting our inclusion criteria for statistical relevance using the Random Forests algorithm, as defined in Materials and methods section. The averaged out-of-bag ‘misclassification errors’ as well as the AUC from the range of the 1000 trees of the Random Forest grown for each of their respective sub-panels are shown in Table 3. We found that the continued ‘focusing’ of the panel from the 14 individual biomarkers to the six-analyte panel improved our ability to correctly classify patients relative to the pathological NSCLC status. However, after the fifth iteration, the AUC and associated sensitivity and specificity values (data not shown) decreased as the number of biomarkers decreased leading us to select the six-member panel from this fifth iteration as the most optimal combination for detecting NSCLC. Individual ‘box and whisker’ plots are shown for these six biomarkers in Figure 1. Next, we defined a classification tree based on a sub-panel of six markers (consisting of TNF-*α*, CYFRA 21.1, IL-1ra, MMP-2, MCP-1, sE-selectin) selected from the Random Forest algorithm within the RPART software package to provide a convenient and useful algorithm for distinguishing NSCLC from benign controls. The classification tree resulting from this process is represented in Figure 2. This tree correctly classified 127 out of the 133 cases (a correct classification rate of 95%). The ROC curve for this classification tree is shown in Figure 3. Test performance characteristics for this panel boast a 97.9% AUC translating to 99% sensitivity and 95% specificity. As reported in our previous studies using this strategy (Borgia *et al*, 2009), we observed a substantial gain in our ability to screen for NSCLC when using the multi-analyte panel over any individual biomarker.

When we validated the performance characteristics of this six-analyte panel that were validated against a second patient cohort, we successfully classified overall 75 of 88 patients. An examination of the individual groups was then performed as a means to confirm the relevance of the associations of the individual biomarkers with promise for the panel to screen for NSCLC. When looking solely at the cohort composed of COPD and asthma patients, only a single patient was misclassified (false positive) of the 40 tested. In the NSCLC cohort, five patients were misclassified of the 33 patients, resulting in an 85% classification rate. Misclassifications were not limited to Stage IA patients, possibly indicating that errors were not due to test sensitivity. And finally, only 8 out of the 15 patients with resected, non-neoplastic disease were correctly classified. This sub-group may require further development in order to improve the range of patients that can be accurately classified by this methodology.

## Discussion

Chest radiography has been widely used historically as a preliminary screening tool because of its wide accessibility, relatively low cost and ease of use. Radiographs, however, have very low specificity and sensitivity when compared with more contemporary imaging techniques such as CT (Ashton and Jett, 2005; Wardwell and Massion, 2005). Therefore, radiography has had very modest success in diagnosing early stage disease. Screening trials have shown that chest radiographs fail to detect 60–80% of early stage lung cancers that were found in the same study by CT (MacMahon *et al*, 2005; McWilliams and Lam, 2005; Mulshine and Sullivan, 2005; Gleeson, 2006). Recent spiral CT advancements have made the method more effective in detecting tumours at a resectable stage than any other modality currently being used for NSCLC. Despite the promising results obtained from the recent spiral CT studies with an increase in early stage disease seen over historical controls, CT screening has not yet been shown to reduce mortality from NSCLC. In addition, CT-screening protocols have several limitations. For example, given the relatively high sensitivity of the technique, coupled with its low specificity, many benign lesions appear as questionable, non-calcified nodules (McWilliams and Lam, 2005; Wardwell and Massion, 2005). These lesions frequently require serial screening to evaluate for growth or more definite neoplastic traits. The interval needed to discern which lesions are neoplastic through serial CT scans may be a critical period in the progression of NSCLC (MacMahon *et al*, 2005; McWilliams and Lam, 2005; Wardwell and Massion, 2005). Therefore, spiral CT is commonly used in combination with a second diagnostic means, such as PET imaging, to attain a more immediate diagnosis. However, the cost of combined imaging modalities may be prohibitive for any widespread screening programmes for early stage disease. Another method routinely used to discern these questionable nodules is the combination of spiral CT with CT-directed fine needle aspirates or bronchoscopy. However, the anxiety and discomfort associated with these invasive techniques make them less than ideal for screening asymptomatic patients. A low cost and minimally invasive serum test would be a much preferred means to complement spiral CT or potentially serve as a pre-screening method to minimise the overall costs of NSCLC detection by better selecting patients to undergo spiral CT. Although no FDA-approved test of this sort currently exists, advancements in the fields of genomics and proteomics bring this screening option closer to reality.

For this study, we used a high-throughput discovery strategy using an extensive arsenal of biomarkers implicated in the literature as having diagnostic and/or prognostic value for NSCLC. The Luminex immunobead platform was important to this strategy, given the ability to process the serum specimens efficiently, whereas using low microliter quantities per panel tested. With the patient cohorts evaluated here, we identified a serum test consisting of TNF-*α*, CYFRA 21.1, IL-1ra, MMP-2, MCP-1 and sE-selectin. The cytokeratin 19 fragment, CYFRA 21.1, is perhaps the most extensively characterised biomarker with diagnostic value for NSCLC (Stieber *et al*, 1993; Bates *et al*, 1997; Molina *et al*, 2003; Tarro *et al*, 2005). Numerous studies have been focused on evaluating its potential for early detection of NSCLC as well as its potential prognostic and predictive value. Each of the remaining analytes has also been previously implicated individually as having either diagnostic value or a function in inflammation, either in NSCLC or other carcinomas. More specifically, TNF-*α*, and IL-1ra are both considered to be acute phase reactants, and as such, they are involved in modulating the immune response and show increased expression in an inflammatory state. Cancer cells are immunogenic and, therefore, lead to the increased expression of proinflammatory agents as well as associated secondary biomarkers. There is an association between chronic inflammation and tumourigenesis, largely because of increases in cell turnover, which can increase serum biomarkers (Yao and Rahman, 2009). Similarly, sE-selectin is a cell adhesion molecule, frequently modulated by inflammation. The MMP-2 is involved in the degradation of proteins in the extracellular matrix during tissue remodelling for epithelial reorganisation.

In terms of performance against the sub-populations within our validation cohorts, our multivariate panel was able to correctly classify most patients with NSCLC as having NSCLC (15% false-negative rate), as well as patients within the Abbott cohort (2.5% false-positive rate) as not having NSCLC. It is difficult to speculate on the possible reasons for the single case of a false positive we observed within Abbott cohort; other than the patient had diagnosed COPD, there was limited clinical data available to us for this patient. However, it is conceivable that this patient may have had undiagnosed or ‘pre-cancerous’ lesions that we detected with our serum test at the time of serum accrual. The sub-population that was the most difficult to classify correctly was the patients with resected non-neoplastic lung disease. Of the patients from this group that were misclassified (47% rate of false positives), all had an inflammatory condition (i.e. pneumonia, pulmonary abscess, hepatitis C) that may have (at least in part) mimicked the biomarker profile that classifies patients as having NSCLC. Symptom severity upon presentation may have elevated these factors in the group that were suspected of having neoplastic lesions relative to patients with a similar set of pathologies, but were exclusively treated in the clinic (such as in the COPD/asthma cohort). With this, future development of the serum test will focus on biomarkers not directly related to inflammation in order to improve specificity of this test and the rate of positive classifications, such as circulating tumour-specific autoantibodies. Given the differences in the measured values across the various groups, it seems unlikely that there was a bias towards patients receiving surgery *vs* those that were treated on an outpatient basis.

Earlier to the report of the panel presented here, the combination of CEA, CA125, CA 19-9, CYFRA 21-1 and NSE was the most efficacious serum test for diagnosing NSCLC, with reported test performance characteristics of a 93.8% sensitivity and 71.5% specificity (Chen *et al*, 2008). Although this panel offers excellent sensitivity, it has poor specificity, making it incapable of serving as a means to complement spiral CT-based-screening protocols and inadequate to serve as a ‘stand-alone’ diagnostic method.

On the basis of the results presented here, we conclude that our NSCLC detection algorithm bases on six serum biomarkers may be a promising low cost and minimally invasive screening test for patients at high risk for NSCLC. Further validation studies are needed to confirm the relevance of this detection algorithm and, ultimately, help bring this much needed screening test into common use. There has also been some consideration of using serum autoantibodies for the early detection of lung cancer (Chapman *et al*, 2008; Tan *et al*, 2009). To further increase the sensitivity and specificity of this panel, the addition of autoantibodies to our present panel is currently in development by our laboratory. We anticipate that the addition of biomarkers of this type may offer the test specificity necessary to discern patients with inflammatory nodules requiring resection from the cases of NSCLC.

## Figures and Tables

**Table 1:** Characteristics of patient populations

	**Discovery**			**Validation**		
	**I**	**II**	**III**	**I**	**II**	**III**
*Age*						
Range	40–83	47–80	46–84	40–80	49–92	46–84
Median	67.5	62	69	61	70	68
						
*Sex*						
Male	3	9	38	6	21	17
Female	9	22	52	9	19	16
						
*NSCLC stage* a						
Ia			16			11
Ib			37			14
IIa			2			2
IIb			11			5
IIIa			19			1
IIIb			5			—
						
*Diagnosis*						
Adenocarcinoma			57			18
Squamous			30			10
Adenosquamous			1			2
NSCLC – other			2			3
						
*Condition*						
No lung pathology		31				
Granuloma	5			6		
COPD				1	34	
Asthma					6	
Sarcoidosis	1					
Pneumonitis	1			1		
Pneumonia	2			1		
Benign cyst	1					
Hamartoma				2		
Chronic inflammation				2		
Lymphoid infiltrate				1		
Thymoma	1					
Lipoma	1			1		

Discovery group refers to the initial group of patients on which 47 biomarkers were tested and multi-analyte panel was created, groups are as follows: I=resected non-neoplastic disease; II=rheumatology controls; III=NSCLC patients. Validation group refers to second cohort on whom our six-multi-analyte panel was tested, groups are as follows: I=resected non-neoplastic disease; II=COPD/asthma patients; III=NSCLC patients.

a Pathologic stage.

**Table 2:** Biomarkers selected for multivariate analysis based on statistical relevance

	**Resected benign nodules (*n*=12)**		**‘Normal’ controls (*n*=31)**		**NSCLC: Stage I^a^** **(*n*=55)**		**NSCLC: Stages II & III^a^,^b^** **(*n*=37)**		**Overall performance characteristics**	
**Biomarker**	**Median^c^**	**Range^c^**	**Median^c^**	**Range^c^**	**Median^c^**	**Range^c^**	**Median^c^**	**Range^c^**	**AUC**	***P*-value^d^**
CYFRA 21-1	371.9	227–783	277.54	122.3–617.2	630.89	169.5–5351	674.56	293.4–3663	0.873	<0.001
TNF-*α*	8.92	3.7–27	4.06	1.37–11.94	14.35	1.37–140	13.85	2.9–41.93	0.862	<0.001
MCP-1	0.47	0.28–1.07	0.77	0.2–2.34	0.40	0.18–2.06	0.4	0.12–5.7	0.753	<0.001
IL-1ra	184.2	42–1168	81.59	2.41–606.4	326.93	2.41–2144	326.93	1.12–3258	0.719	<0.001
MMP-2	6792	4440–10013	7757	5029–12836	6195	3293–12693	5516	897–10094	0.705	<0.001
IL-6	37.59	1.17–1495	11.39	1.17–1520	61.46	1.17–5862	54.99	3.44–906.5	0.702	<0.001
EOTAXIN	0.135	0.07–0.23	0.16	0.06–0.26	0.10	0.04–0.39	0.11	0.04–0.56	0.698	<0.001
CA-125	1.13	0.43–11.47	0.57	0.12–4.92	1.38	0.12–143.4	1.8	0.1–24.6	0.698	<0.001
sE-selectin	1643	702–3962	1635	862–4079	1198	417–2603	1283	509–2547	0.690	<0.001
sP-selectin	2779	1265–8764	3168	1722–5158	2726	926–5181	1892	926–11835	0.677	<0.001
MIP-1*α*	0.13	0.1–0.15	0.14	0.12–0.21	0.13	0.1–0.7	0.12	0.05–0.81	0.669	0.00117
IL-10	21.32	3.04–92.08	3.04	3.04–576.6	16.83	3.04–1361	42.5	3.04–428	0.667	0.00162
CRP	2.659	0.945–4.336	4.89	0.945–23.7	2.69	0.95–10.2	3.05	0.015–9.92	0.662	0.00245
IL-2R*α*	54.4	18.7–119.7	29.815	5.33–224.62	57.44	5.33–3359	46.98	7.03–192.8	0.652	0.00462

a Pathologic stage.

b Lymph node-positive disease.

c Values expressed as pg ml^–1^.

d Mann–Whitney *U* (two-sided test).

Descriptive statistical parameters and individual test performance characteristics measured from our training cohort for each biomarker within our statistical thresholds.

Abbreviations: NSCLC=non-small cell lung cancer; AUC=area under the curve; TNF=tumour necrosis factor; MCP=monocyte chemotactic protein; IL=interleukin; MMP=matrix metalloproteinases; CA=cancer antigen; MIP=macrophage inflammatory protein; CRP=C-reactive protein; CYFRA=CYFRA 21-1, cytokeratin 19 fragment.

**Table 3:** Variable selection of biomarkers using Random Forests

		**Biomarkers**															
	**Variables**	**MIP-1*α***	**SCF**	**CEA**	**TNF-RI**	**TNF-*α***	**IFN-*γ***	**M-CSF**	**G-CSF**	**TNF-RII**	**sICAM-1**	**MMP-2**	**CRP**	**IL-2R*α***	**Osteopontin**	**IL-1ra**	**OOB**
1	15	X	X	X	X	X	X	X	X	X	X	X	X	X	X	X	0.336
2	12	X	X	X	X	X	X	X	X	X	X	X	X				0.308
3	10	X	X	X	X	X	X	X	X	X	X						0.308
4	8	X	X	X	X	X	X	X	X								0.317
5	6	X	X	X	X	X	X										0.308
6	5	X	X	X	X	X											0.289
7	4	X	X	X	X												0.345

Abbreviations: OOB=out-of-bag (misclassification error); TNF=tumour necrosis factor; IL=interleukin; MMP=matrix metalloproteinases; MIP=macrophage inflammatory protein; CRP=C-reactive protein; SCF=stem cell factor; IFN=interferon; G-CSF=granulocyte colony-stimulating factor; M-CSF=monocyte colony-stimulating factor; sICAM-1=soluble intracellular adhesion molecule 1.
